# Numerical Simulations of Strength Characteristics of Lightweight Fibre-Reinforced Concrete

**DOI:** 10.3390/ma19102121

**Published:** 2026-05-18

**Authors:** Agnieszka Michalik, Łukasz Gołębiowski, Filip Chyliński

**Affiliations:** Building Research Institute, Building Structures, Geotechnics and Concrete Department, ul. Filtrowa 1, 00-611 Warsaw, Poland; l.golebiowski@itb.pl (Ł.G.); f.chylinski@itb.pl (F.C.)

**Keywords:** lightweight aggregate concrete, steel fibres, fibre-reinforced concrete (FRC), tensile strength, fibre-reinforced concrete model, microstructure, Finite Elements Method (FEM), concrete hardening/softening, Menetrey–Willam model

## Abstract

Low tensile strength (brittleness) is a significant drawback of lightweight aggregate concrete, as it significantly limits its application. The parameters can be improved by using dispersed reinforcement. For the purpose of the study, two fractions of high-strength lightweight aggregate were used. It was produced by sintering waste material from power plants and cogeneration plants (e.g., fly ash). Hook-shaped steel fibres were used as the reinforcement. The presented tension test results apply to lightweight fibre-reinforced concrete, i.e., flexural tensile strength, splitting tensile strength and residual flexural tensile strength compared to lightweight non-reinforced concrete. It also refers to the analysis of fibre distribution using computer tomography and the microstructure of the fibre–cement slurry contact zone. The test results revealed that steel fibres are distributed correctly in lightweight concrete, creating effective reinforcement for the brittle cement matrix. The experimental work was supported by numerical simulations based on the Finite Element Method (FEM). A lightweight concrete structure with volumetric content and steel fibre distribution identical to those used in the experiment was modelled. This way, the numerical simulations were verified. The confirmation of the numerical model’s reliability shall help engineers develop the material’s strength at the product design stage. The optimisation shall be possible owing to the easy application of the fibres’ variable configuration, given their share and orientation. As a result of combining experimental tests with numerical simulations, the paper evaluates the influence of steel fibres on the strength of lightweight concrete. Ansys Workbench software was used to model a three-point bending test on lightweight concrete beams. A Menetrey–Willam constitutive model was selected to represent the mechanical behaviour of fibre-reinforced concrete; the model assumed material hardening/softening. Simulations yielded numerical responses similar to the experimental results, confirming the model’s ability to capture the fibre reinforcement’s influence on the forms of destruction.

## 1. Introduction

Plain concrete is the most popular construction material. That is why lightweight aggregate concrete is often perceived as a material primarily characterised by low density and limited suitability for construction, which limits its use to thermal and sound insulation. Owing to the development of high-strength lightweight aggregates, cements, and chemical admixtures, modern lightweight aggregate concrete can be successfully used as a valuable construction material. Due to its potential to achieve high strength values (>60 MPa) at a lower weight (low density), lightweight aggregate concrete can be widely used for prefabrication and monolithic construction [1,2,3,4], as contemporary infrastructure faces increasing demand for quick and economical construction. The primary advantages of LWAC (lightweight aggregate concrete) compared to plain concrete include dead load reduction and a high strength-to-weight ratio. It is beneficial for the construction of overpasses, long-span bridges, floating platforms, and high-rise buildings, where the dead load of plain concrete is a significant part of the computational load [1,5,6]. By reducing the structure’s load, lightweight concrete also reduces the designed loads of foundations and ceilings, which can help decrease the costs. LWAC is also employed in the production of items such as chimney blocks, ventilation blocks, hollow masonry units, planters, and palisades. LWAC can be helpful as thermal insulation for basement and foundation walls and as a concrete floor layer (it fulfils two roles: that of a concrete substrate and thermal insulation) [2,3,4,7,8,9,10].

A literature review revealed that the brittleness of lightweight aggregate concrete limits its use [11]. The material is characterised by low tensile strength, which prevents its broad application in construction. Flaws related to brittleness can be eliminated by introducing dispersed steel fibre reinforcement, which will increase the LWAC cracking energy. Various types of steel fibres are expected to be used. They include recycled fibres, e.g., car tyres, whose effectiveness was confirmed for plain concrete [12,13]. The modification of sintered lightweight aggregate concrete shall extend its application in civil engineering.

High-strength lightweight aggregate created by sintering waste material from power plants and cogeneration plants (e.g., fly ash) is used in this study [14]. This kind of aggregate helps to produce high-strength (>60 MPa) lightweight concrete. The use of lightweight aggregate from waste material aligns with sustainable construction policy, which aims to conserve natural resources and maximise the use of recycled materials [14]. Lightweight aggregate is usually produced from industrial waste (fly ash), which qualifies it for a circular economy. The aggregate is made from anthropogenic minerals (fly ash from power plants), which means it utilises industrial waste. Studies on the use of lightweight aggregate in construction concrete are multidimensional [15,16,17,18,19,20,21,22,23,24].

The study shall broaden knowledge of technology and further optimise the mechanical characteristics of lightweight aggregate fibre-reinforced concrete [1,3,20,24] by introducing numerical simulations previously not described in the literature. The previously analysed fibre-reinforced concrete was tested with standard methods. Confirming the reliability of the developed numerical model of steel fibre-reinforced concrete is an innovative aspect of this paper. The research problem involves assessing the optimisation of lightweight fibre-reinforced concrete using numerical simulations. This work modelled the macro-scale structure of a steel fibre-reinforced composite with a non-rectilinear shape and irregular distribution within the concrete matrix. The preliminary FEM analyses presented in this work will be continued in the future. It is planned to develop more advanced models at a smaller scale, where the representative model will cover a much smaller sample section. This will enable a more accurate representation of the composite structure by taking into account the fibre surface and the contact between the fibres and the matrix. The results obtained at the smaller scale will then be imported to the macro-scale. The developed model shall help continue numerical work on the optimal distribution and content of various types of fibres for specific construction needs.

The aim of this work is to use computer simulations in the design of lightweight concrete reinforced with steel fibres. The numerical work is built upon and verified against experimental observations. The contribution of this work is an experiment-supported numerical modelling study.

Concrete is a complex and non-uniform material characterised by non-linear behaviour, diversified tensile and compressive strength, and shear expansion [25]. Moreover, in order to improve a structure’s strength, producers use fibres or parts of other materials in the concrete mix. Hence, when designing the structure of concrete, it is crucial to select the right numerical tools and constitutive models representing the material’s behaviour.

The majority of constitutive concrete models are based on a standard theory of plasticity. The key components of any model based on a theory of plasticity include the yield criterion, the flow rule, and the hardening rule, interpreted so that it explains material hardening and softening and can be identified with the equations of internal variable evolution included in the yield criterion.

Constitutive relations of a classic theory of plasticity are broadly used in a non-linear simulation of reinforced concrete structures. A constitutive Menetrey–Willam (MW) model is among the most popular concrete models [26,27,28] used in ANSYS 2023/R1 software. The MW constitutive model predicts the mechanical behaviour of concrete under complex stress conditions, including not only simple tension and compression but also combined tension, compression, shear, and tri-axial conditions. The MW model is also suitable for representing fibre-reinforced concrete in general situations, while dispersed cracking and damage models focus more on capturing the progressive degradation and cracking whose effects are macroscopically alleviated by the presence of steel reinforcement.

The MW model was used in [29] to design polymer-fibre-reinforced concrete and to determine the residual tensile strength. The authors of [30] used the MW plasticity surface to consider the non-linear behaviour of aggregate and cement slurry in analysing the cracking of concrete containing steel fibres. Numerical modelling using the Menetrey–Willam plasticity surface, which considers concrete behaviour under compressive and tensile stress, is an essential tool for better understanding fibre-reinforced concrete behaviour.

## 2. Materials and Methods

### 2.1. Materials

In this study, two fractions (1–4 and 4–9) of artificial green lightweight aggregate were used for lightweight aggregate concrete. The aggregate was formed by sintering waste material from power plants and cogeneration plants, e.g., fly ash. Artificial lightweight aggregate is produced using waste-free technology, and sintering is an autothermal process—fuel from an external source is required only to initiate sintering. Process heat (flue gas up to 1000 °C) is recovered and partially used in production. The produced lightweight aggregate is an environmentally friendly product whose valuable characteristics, such as durability, strength and low weight, determine its further use as aggregate for lightweight concrete in lightweight prefabricated construction elements, thermal insulation layers, road construction, geotechnics and gardening (drainage layers).

**Figure 1:**
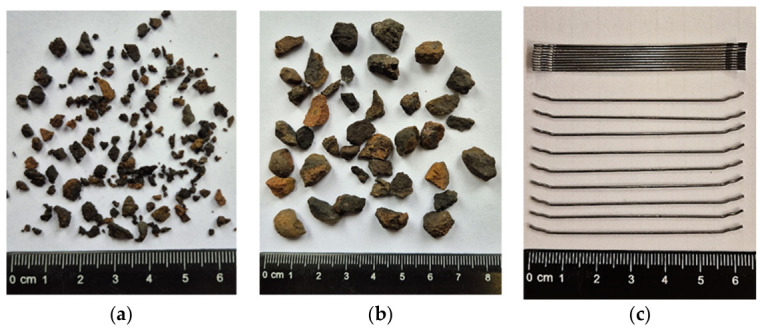
Ingredients of lightweight aggregate fibre-reinforced concrete: (**a**) lightweight aggregate 1–4, (**b**) lightweight aggregate 4–9, (**c**) steel fibres.

Figure 1a,b show two fractions of lightweight aggregate (LSA, CERTYD, Poland). Table 1 and Table 2 summarise the characteristics of the lightweight aggregate used in this study.

Hook-shaped steel fibres (diameter: 0.75 mm, length: 60 mm, slenderness (length to diameter ratio): 80) were used as dispersed reinforcement. The fibre length, based on the manufacturer’s declaration (Bekaert, Zwevegem, Belgium), was 60 mm in projection. The fibres had a hooked shape and an unrolled length of approximately 70 mm. Furthermore, the actual average fibre length based on computed tomography was 70 mm, so 70 mm was assumed in the numerical model. The fibres are characterised by the tensile strength of 2200 MPa and Young’s modulus of 210 GPa.

### 2.2. Lightweight Concrete Formula

Table 3 summarises the designed lightweight concrete formula with steel fibres. The concrete was reinforced with steel fibres to improve resistance to brittle cracking. Hook-shaped steel fibres, 50–60 mm long, are typically used for concrete reinforcement.

The following symbols were used in this paper to describe concrete:

- LK—lightweight control concrete, according to the formula in Table 1, with no fibres;
- LW—lightweight concrete with fibres, according to the formula in Table 1.

### 2.3. Methods

The following tests were performed on concrete mixes and hardened lightweight concrete with and without fibres.

#### 2.3.1. Testing the Concrete Mix Characteristics

In order to evaluate the influence of fibre addition on the characteristics of aggregate-based concrete mix, the following tests were performed:

- Determination of concrete mix consistency by the cone fall method according to EN 12350-2:2019 [31]
- Air content in the mix—pressure method according to EN 12350-7:2019 [32]
- Concrete mix density according to EN 12350-6:2019 [33]

#### 2.3.2. Testing the Strength Characteristics of Lightweight Concrete

In order to evaluate the influence of fibre additive on the strength and functional characteristics of lightweight concrete, the following tests were performed:

- Cured concrete absorbability according to PN-B-06250:1988 [34]
- Compressive strength after 28 days according to EN 12390-3:2019 [35]
- Flexural tensile strength after 28 days according to EN 12390-5:2019 [36]
- Splitting tensile strength after 28 days according to EN 12390-6:2024 [37]
- Concrete’s modulus of elasticity according to EN 12390-13:2021 [38]
- Residual flexural tensile strength according to EN 14651+A1:2007 [39].

#### 2.3.3. Computer Tomography Analysis of Fibre Distribution

The distribution of fibres in the concrete was analysed for 100 × 100 × 100 mm cubic specimens made of lightweight concrete. The specimens contained 20 kg of fibres per m^3^. The test was performed to observe fibre distribution in hardened lightweight concrete and to check if the fibres are uniformly distributed in the mix during mixing. The test was carried out using a General Electric (GE V|TOME|X M300) computer tomography scanner (manufacturer: GE Sensing & Inspection Technologies GmbH (abbreviated as GE), Wunstorf, Germany, 2019).

#### 2.3.4. Microstructure Analysis

The microstructure tests aimed to assess the microstructure of lightweight concrete, with particular attention to the contact zone between the lightweight aggregate and the cement slurry. The other objective was to evaluate the microstructure of lightweight concrete containing steel fibres by analysing the fibre–cement slurry contact zone. Additionally, it was vital to analyse the content of air voids in the steel fibres’ contact zone to make some assumptions for the material model.

The specimens for microscopic tests were prepared by cutting ca. 5 mm thick slices from six cubic samples with an edge length of 100 mm. Specimens with ca. 20 mm × 20 mm were then cut out from the central parts of the slices. The small specimens were dried, resin-coated under low pressure and then ground and polished in numerous cycles. The details of specimen preparation for microscopic tests are described in a previous publication [40].

Microscopic tests were carried out in two stages, i.e., optical and scanning microscopy. A stereoscopic optical microscope from ZEISS Stemi 508 (Carl Zeiss Microscopy GmbH, Köln, Germany) was used for the observations under reflected light. The scanning microscopy analyses were performed with a ZEISS Sigma 500 VP device (Carl Zeiss Microscopy GmbH, Köln, Germany). BSD images were recorded. The elemental composition of a microarea was analysed, and an element distribution map was developed using an EDX detector UltimMax 40 (Oxford Instruments, High Wycombe, UK). The polished section specimens were evaporated with gold before the tests.

Pore size distribution was analysed using statistical image analysis software ZEN (Blue Edition ver. 3.3.89.0000 by ZEISS). An area of approximately 500 mm^2^ of polished section was analysed for each concrete. Analysis was performed only in the area of cement matrix avoiding areas of porous aggregate grains. Due to the resolution of the analysed images and magnification during collection, the smallest pore diameter taken into account is 2.25 µm.

#### 2.3.5. FEM Numerical Simulations According to the Menetrey–Willam Constitutive Model

A three-point bending test on 150 mm × 150 mm × 550 mm rectangular notched concrete beams was selected for numerical simulations. Flexural strength was determined and compared for the following two types of beams: (1) LK made of lightweight concrete, and (2) LW made of lightweight concrete with steel fibres. The ANSYS 2023/R1 software with a non-linear material and geometrical analysis was used for the calculations. The Menetrey–Willam model was assumed for concrete. The model considers the material’s hardening/softening under single-axial stress. An elastic–plastic material model was assumed for steel fibres. An incremental iterative solution was also used in the calculations, employing the Newton–Raphson method. The numerical simulations aimed to develop a computational model of concrete reinforced with steel fibres.

## 3. Results and Discussion

### 3.1. Concrete Mix Characteristics

Table 4 summarises the properties of the LK (lightweight control concrete) and LW (lightweight concrete with fibres) concrete mixes.

According to Table 4, adding 20 kg of steel fibres per m^3^ resulted in a minor decrease in workability (smaller slump loss) and a higher mix density.

### 3.2. Strength Properties of Hardened Lightweight Concrete

Table 5 summarises the test results for hardened LK and LW concrete after 28 days.

**Figure 2:**
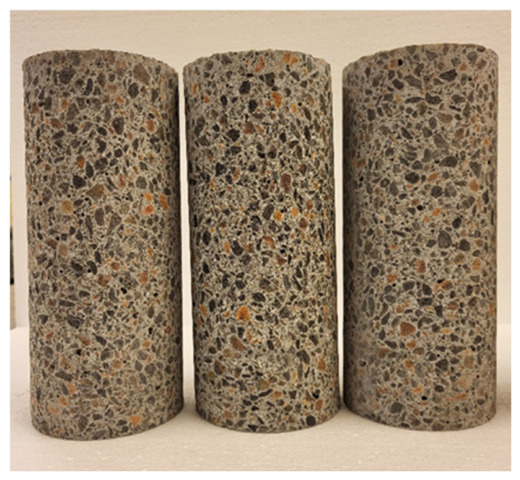
Specimens for testing the modulus of elasticity.

**Figure 3:**
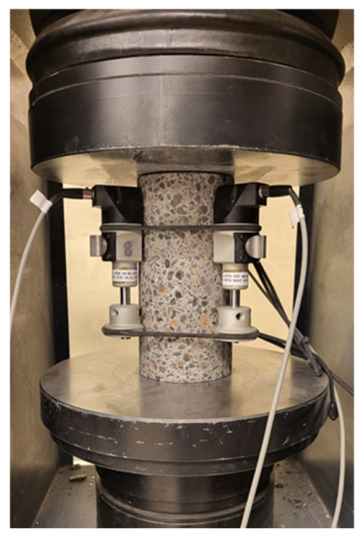
Testing the modulus of elasticity.

The number of samples for the studies presented in Table 5 is 6, and the results are presented as the mean with expanded uncertainty. Differences smaller than the measurement uncertainty were considered insignificant. However, taking into account the simple acceptance rule, the results of lightweight concrete tests indicate that the addition of 20 kg/m^3^ of steel fibres to lightweight concrete increases compressive strength by approximately 5%. Both LK and LW concretes meet the requirements for compressive strength class LC40/44 according to 206+A2:2021-08 [41]. For the flexural strength test, LW concrete achieved approximately 4% higher flexural strength than LK concrete, which opens up prospects for the use of LW concrete in structural concrete. The results of the elastic modulus tests for LK and LW concretes are similar (Figure 2 and Figure 3). These are quite high values for lightweight concrete; the modulus for normal concrete is around 20 GPa. The splitting tensile strength of LW and LK concretes reached the same values.

**Figure 4:**
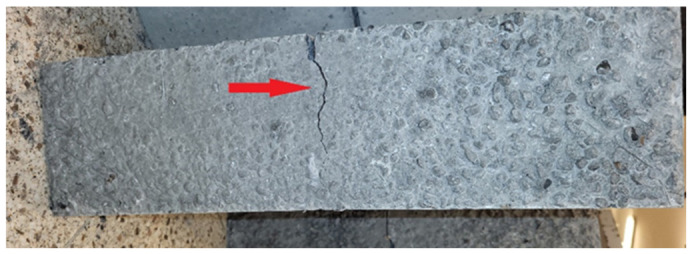
Lightweight concrete specimen after residual flexural tensile strength testing. The arrow marks the incision and specimen crack.

The residual flexural tensile strength tests were performed after 28 days according to EN 14651+A1:2007 [39] only for the LW concrete (with fibres). Figure 4 shows a 150 mm × 150 mm × 550 mm concrete beam with an incision that is the specimen crack propagation area.

**Figure 5:**
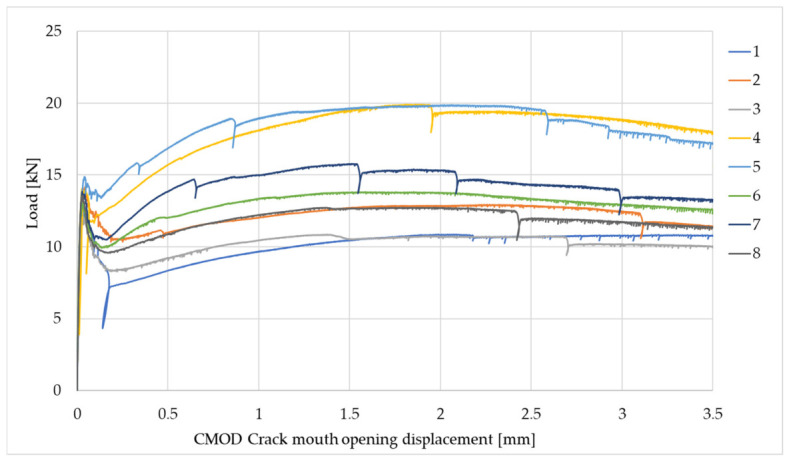
Load tests—CMOD for the LW concrete.

Table 6 and Figure 5 show the results of residual flexural tensile strength tests.

Residual strength testing is the primary measure of the efficacy of lightweight concrete reinforcement. Further loads are transferred over the cement matrix by fibres. All specimens of lightweight concrete reinforced with steel fibres transferred the loads. The test results revealed high residual flexural tensile strength, confirming the effectiveness (efficacy) of steel fibre reinforcement in lightweight concrete. The results of a flexural tensile strength test are primarily influenced by the fibre distribution at the centre of the concrete beam at the notch. After the concrete fractures at the notch, mid-span, the load is transferred through the fibres, i.e., through the fibre–concrete interaction. Therefore, the residual strength results usually exhibit some scatter. In the presented tests, all specimens withstood the loads until the end of the test, indicating good fibre distribution and adhesion to concrete. The variability in the results in this test is acceptable and the test result is an average.

### 3.3. Fibre Distribution Analysis Using Computer Tomography

**Figure 6:**
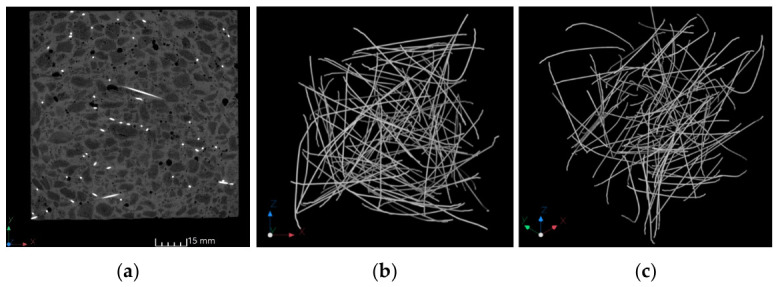
Fibre distribution images generated using computer tomography: (**a**) cross-section through the sample, (**b**,**c**) 3D view of the fibre distribution.

Figure 6 shows the results of fibre distribution tests using computer tomography.

CT-generated images revealed that the steel fibres are uniformly distributed in concrete. Fibre clusters are not formed. Owing to the uniform distribution, the fibre reinforcement efficacy is high.

### 3.4. Microstructural Analysis

LK—lightweight concrete with no fibres.

**Figure 7:**
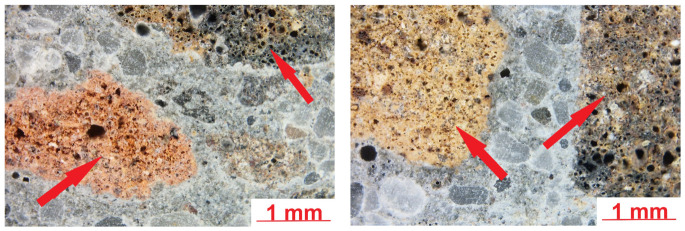
Microstructure of LK lightweight concrete; lightweight aggregate grains are marked with arrows (optical microscopy).

Figure 7 shows sample images of the LK lightweight concrete microstructure.

**Figure 8:**
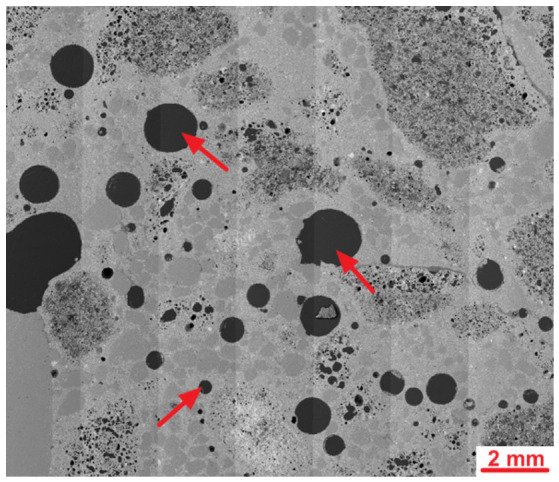
SEM images of the LK concrete microstructure; air pores are marked with arrows.

The microstructure of the lightweight concrete was analysed using an optical microscope. It revealed the presence of fine and coarse lightweight aggregate grains in the cement slurry. Lightweight aggregate grains were characterised by higher porosity. The cement slurry–lightweight aggregate contact zone at this magnification level was mostly tight and compact. Air macropores were observed in the cement slurry. Further analyses were carried out using a scanning microscope. The SEM images of the analysed specimen surface are shown in Figure 8.

**Figure 9:**
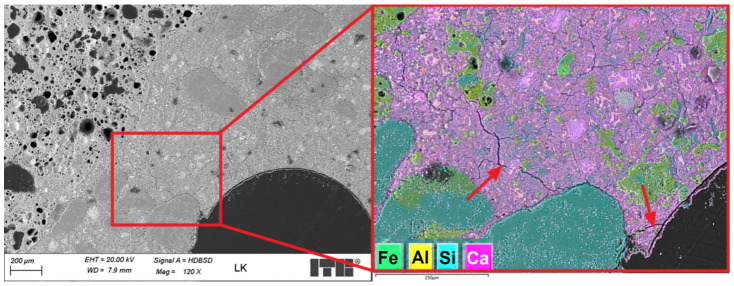
LK concrete microstructure; shrinkage cracks are marked with arrows.

An SEM analysis of the LK specimen’s microstructure revealed the presence of mostly spherical air macropores in the cement slurry. The diameters of the largest pores exceeded 2 mm. The high content of spherical macropores resulted from the intended aeration of the cement slurry to make the concrete freeze-proof. The concrete was designed to be frost-resistant, but test results regarding the frost resistance of lightweight concrete with fibres will be the subject of the next article. Figure 9 shows a sample microstructure image recorded during further studies.

**Figure 10:**
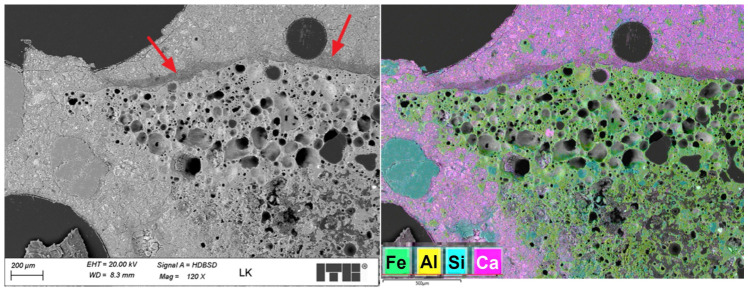
Microstructure of LK concrete; areas with a different contact zone structure are marked with arrows.

A further analysis of the LK concrete microstructure revealed the presence of Portland cement clinker relics in the cement slurry alongside fine grains of porous aggregate, which could have originated from the applied aggregate or were formed during concrete mixing. The formed cement matrix was tight and compact. Shrinkage microcracks were observed in the cement matrix most likely due to the relatively high cement content of the concrete mix. Those microcracks should not affect the durability. The contact zone between the cement slurry and the non-porous quartz aggregate was compact and tight. An analysis of the contact zone between the cement slurry and the porous aggregate grains revealed greater diversity, as shown in the example in Figure 10.

The microstructure of the contact zone between the porous aggregate and the cement slurry was mainly tight and compact. Neither excess migration of calcium ions into the aggregate structure nor water release from the aggregate to the slurry was observed. It was manifested by a lower calcium concentration in the contact zone. This proves the aggregate’s correct initial saturation with water. In some areas, lower calcium content was observed in the contact zone alongside increased porosity of some aggregate surfaces oriented in the same direction (Figure 10). The observed changes were most likely water lenses, which can be seen in reverse in Figure 10, occurring below the aggregate grains. This effect contributes to a local increase in the water–cement ratio and softening of the contact zone. It should be emphasised that the phenomenon was observed only in some aggregate grains and should not have any major significance for the composite’s strength or durability characteristics.

LW—lightweight concrete with fibres.

**Figure 11:**
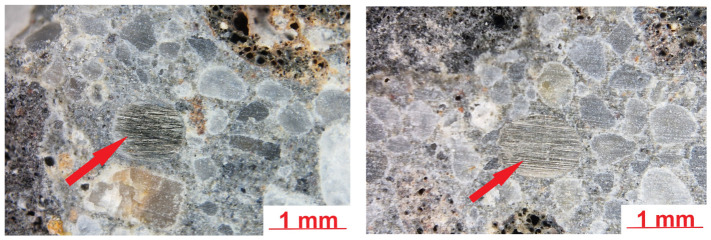
LW concrete specimen microstructure; steel fibres are marked with arrows (optical microscope images).

Figure 11 shows sample microstructure images of a lightweight concrete specimen containing steel fibres. The images were taken using an optical microscope.

**Figure 12:**
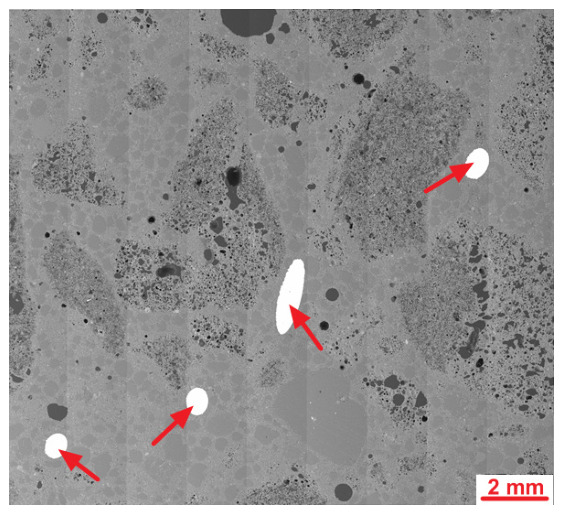
SEM images of the analysed LW concrete specimen; steel fibres are marked with arrows.

The microstructure observations of lightweight concrete with steel fibres (LW) using an optical microscope revealed a structure similar to that of the reference concrete with no steel fibres (LK) in the areas with no fibres. The observed steel fibres were well embedded in the cement matrix, and no significant discontinuities were detected in the contact zone at this magnification level. The steel fibres were relatively uniformly distributed in the concrete, with no tendency to clustering. The addition of steel fibres was not found to increase the number of macropores in the slurry—the observed effect was the opposite. Figure 12 shows SEM images of the analysed surface of the LW specimen.

**Figure 13:**
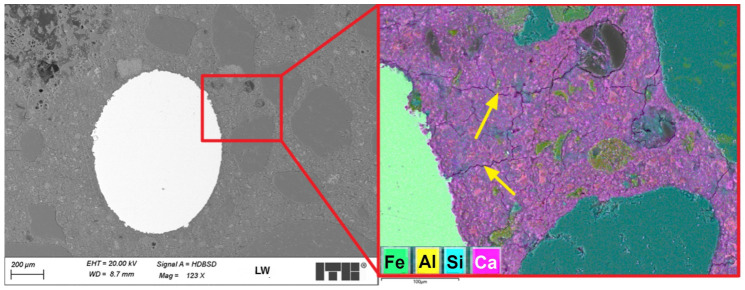
LW concrete microstructure; cracks propagating primarily in the direction perpendicular to the steel fibre are marked with arrows.

An SEM microstructure analysis of the LW concrete revealed a microstructure similar to that observed in the LK specimen. Much fewer air macropores were detected than in the LK specimen, which is untypical. According to the literature, the addition of fibres can increase the air content in plain concrete [41]. The steel fibres in the cement matrix were covered entirely with slurry with no apparent discontinuity or air voids. Figure 13 shows a sample image recorded during an analysis of a fibre–cement slurry contact zone.

An analysis of the LW concrete specimen microstructure in the fibre–cement slurry contact zone revealed that the structure of the steel fibres was developed and that the pits were filled with slurry. Such a development in the contact zone positively affects the adhesion of fibres and improves the composite’s strength characteristics. Shrinkage cracks in the cement matrix were also observed in the steel fibres’ surroundings. Some of the observed microcracks were initiated on rough fibre surfaces (Figure 13), but the fibres were found to limit the propagation of larger cracks. The contact zone was assessed based on a comparison to previous studies where pull-out tests of fibres from the cement matrix were also performed [13].

**Pore size and distribution analysis**.

Table 7 presents the results of the pore size distribution analysis performed using statistical image analysis.

An analysis of the pore size distribution results revealed that adding fibres to concrete reduces the total porosity of the cement matrix. However, the number of pores increased. It can therefore be concluded that adding fibres to lightweight concrete is beneficial because it promotes the formation of a greater number of smaller air voids, which positively affects the frost resistance of the cement matrix and the concrete as a whole.

### 3.5. Numerical Simulation of Concrete with Steel Fibres

#### 3.5.1. Menetrey–Willam Constitutive Model

**Figure 14:**
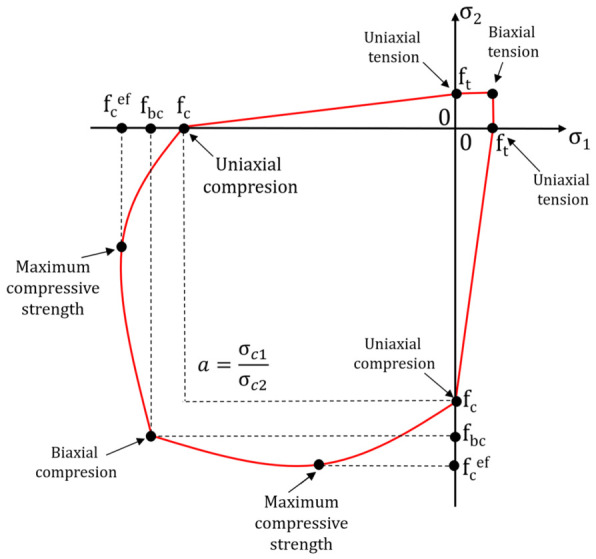
William–Warnke plasticity area for a flat stress condition.

The Menetrey–Willam constitutive model contains parameters that help reconstruct the physical non-linearity and the material’s permanent strain under tensile and compressive stress. The Menetrey–Willam (MW) constitutive model assumes material isotropic hardening/softening described by the William–Warnke plasticity areas [26] for a flat stress condition (Figure 9). The area is expressed with the main stresses σ1 and σ2, and the material parameters f_t_, f_c_, and f_cb_, which represent the limit strengths under uniaxial tension, uniaxial compression, biaxial tension, uniaxial compression, and biaxial compression of concrete, respectively. The MW area is also formed by the counterpart of the f_c_^ef^ concrete compressive strength, taking the maximum stress value (Figure 14). The parameter can be determined based on the Kupfer criterion [42]. The criterion was estimated based on experimental tests on concrete under biaxial compression. The counterpart of the compressive strength was expressed with the following formula:

$$f_{c}^{ef}=\frac{1+3.65a}{{\left(1+a\right)}^{2}}f_{c}$$

where: a = σ_c1_/σ_c2_, and σ_c1_ and σ_c2_ are the primary compressive stresses and f_c_ is the material strength under uniaxial compression.

Concrete behaviour during hardening/softening is modelled by changing the load area size and permanent strain area. The area sizes in the primary stress space depend on the current strength values that are determined by the Ω_t_ and Ω_c_ hardening/softening functions for compression and tension, respectively:

$$\Omega_{tc}=\frac{\sigma_{t}}{f_{ct}}$$

$$\Omega_{c}=\frac{\sigma_{c}}{f_{c}}$$

where: *f*_*c*_ and *f*_*t*_ denote uniaxial compression and uniaxial tension, respectively.

**Figure 15:**
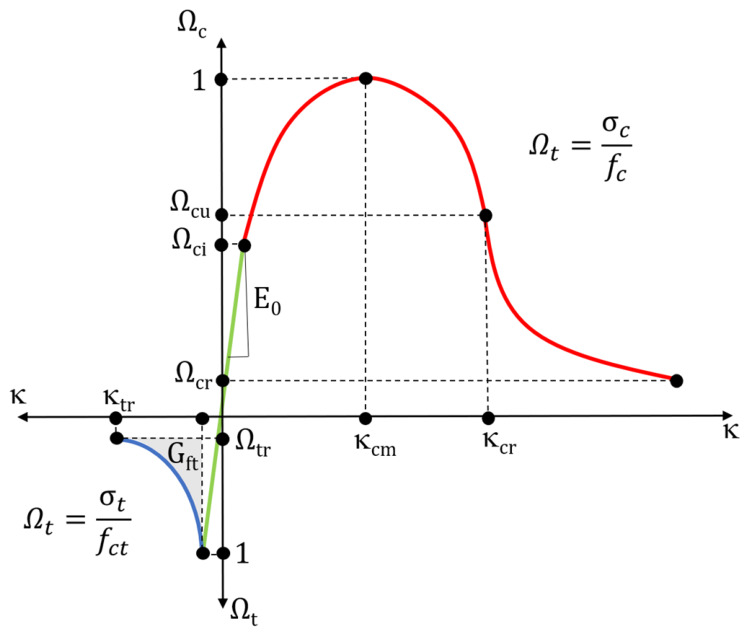
Menetrey–Willam concrete hardening/softening model under uniaxial concrete stress at compression and tension.

The Ω_t_ and Ω_c_ hardening and softening functions evolve as K_c_ and K_t_ permanent strain occurs under uniaxial compression and tension. The load surface behaviour during hardening/softening under uniaxial stress according to the Menetrey–Willam model is shown in Figure 15.

Before permanent strain occurs under uniaxial compression, the Ωc hardening function follows a linear relationship until it reaches the maximum value of the Ωc relative stress, which also marks the onset of non-linear hardening. The hardening function of permanent strain κ_c_ < κ_cm_ can be expressed with the following formula:

$$\Omega_{c}=\Omega_{ci}+(1-\Omega_{ci})\times \sqrt{2\times \frac{\kappa_{c}}{\kappa_{cm}}-\frac{\kappa_{c}^{2}}{\kappa_{cm}^{2}}}$$

where K_cm_ is the plastic strain at uniaxial compressive strength.

For permanent strain κ_c_ = κ_cm_, the compressive strength is reached, and softening begins. There are two softening scenarios under uniaxial compression:

- For permanent strain κ_cm_ < κ < κ_cr_:

$$\Omega_{c}=1-\frac{1-\Omega_{cr}}{\kappa_{cr}-\kappa_{cm}}\times (\kappa_{c}-\kappa_{cm})$$

where Kcr is the maximum effective plastic strain under uniaxial compression, and Ω_cr_ is a relative residual compressive stress

- For permanent strain κ > κ_cr_:

$$\Omega_{c}=\Omega_{cr}+\left(\Omega_{cu}-\Omega_{cr}\right)\times exp\left(2\times \frac{\Omega_{cu}-1}{\kappa_{cr}-\kappa_{cm}}\times \frac{\kappa_{c}-\kappa_{cr}}{\Omega_{cu}-\Omega_{cr}}\right)$$

where Ω_cr_ is a relative residual stress of the compressed concrete.

For concrete exposed to tension, the model uses a generalised Hooke’s law until the f_ct_ maximum stress is achieved. After exceeding the f_ct_, stress is released and reaches the residual tensile compressive stress (Ω_tr_), corresponding to the tensile plastic strain limit (**κ**_tr_).

Under further strain, the material is considered fully unsuitable for transferring loads. G_ft_ is the energy of concrete cracking under tension.

#### 3.5.2. Numerical Model of Three-Point Bending of Concrete Beams Containing Fibres

A three-point bending test was selected for numerical simulations. It was performed on 150 × 150 × 550 mm notched concrete beams. The geometry and loading pattern of the beams were reconstructed based on three-point bending in laboratory conditions. The experimental test is described in Item 3.5.1.

**Figure 16:**
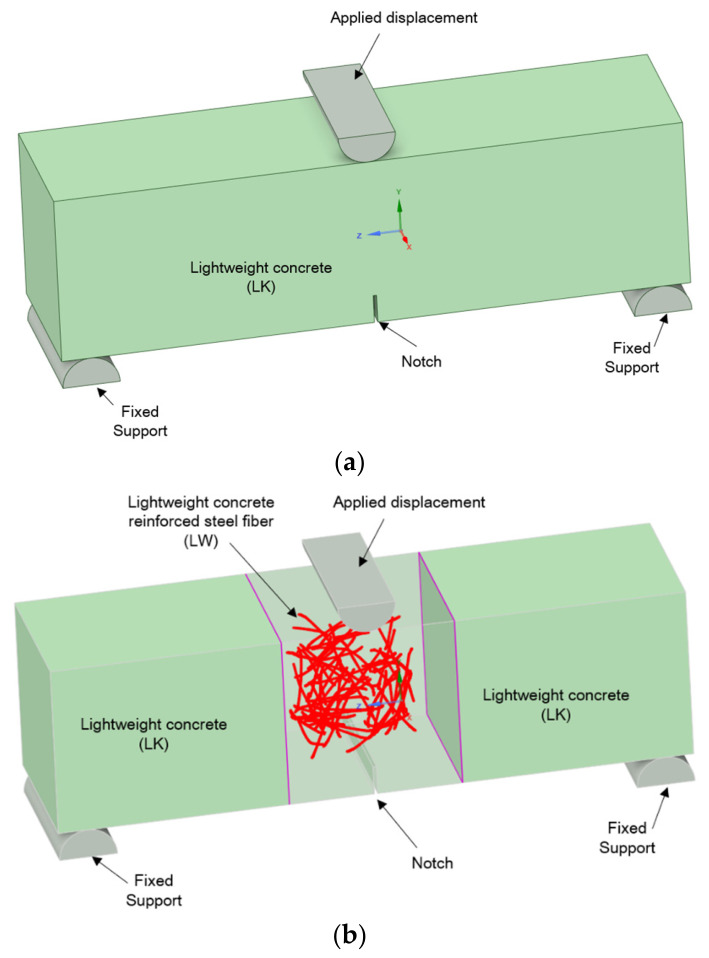
Beam models for three-point bending numerical analysis: (**a**) Model 1 of LK concrete, (**b**) Model 2 of LW concrete reinforced with fibres.

**Figure 17:**
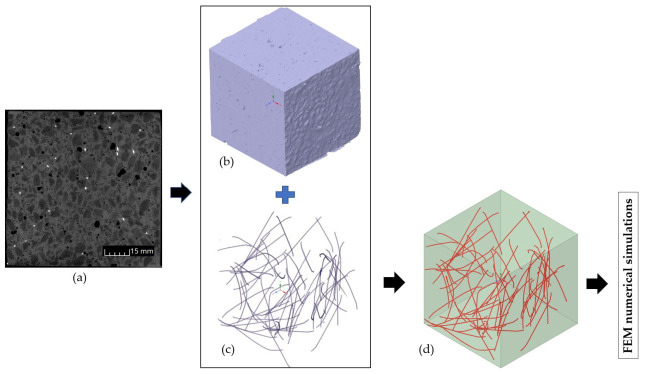
Workflow of generating the LW concrete structure’s 3D model: (**a**) CT flat photo, (**b**) 3D model of concrete matrix, (**c**) 3D model of steel reinforcement, (**d**) 3D CAD model.

Beam 1 was modelled using uniform primary material—LK lightweight concrete (Figure 16a). In beam 2, dispersed steel fibres were additionally distributed as a line in the primary material (Figure 16b). 70 mm long fibres with a 20 kg/m^3^ share in the material volume were oriented in the same way as in the physical sample described in Item 3.1. The fibre length, based on the manufacturer’s declaration, was 60 mm in projection. The fibres had a hooked shape and an unrolled length of approximately 70 mm. Furthermore, the actual average fibre length based on computed tomography was 70 mm, so 70 mm was assumed in the numerical model. This has now been corrected in the article. Fibre distribution was represented using computer tomography and graphic design software, enabling the representation of spatial geometry (Figure 17). The 0.75 mm diameter and “reinforcement” attribute were assigned to the linear finite elements of the fibres.

A perfect connection was assumed for the contact point between the reinforcement and the concrete. An incremental displacement of 0.2 mm downwards was used at the load application point.

Concrete characteristics were assumed based on the Menetrey–Willam constitutive model, considering hardening/softening under uniaxial stress. The material characteristics of lightweight concrete and steel are summarised in Table 8 and Table 9.

**Figure 18:**
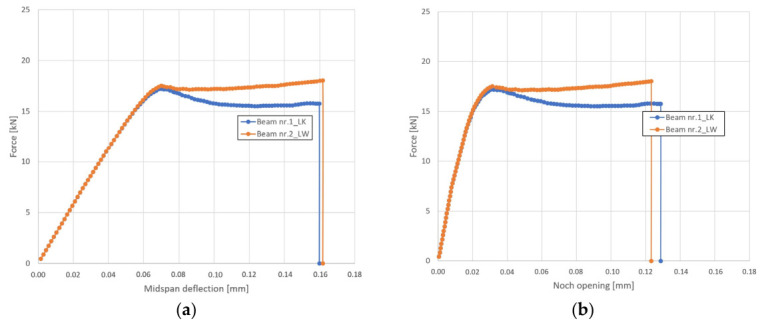
Flexural strength curves: (**a**) force–deflection; (**b**) force–notch opening.

**Figure 19:**
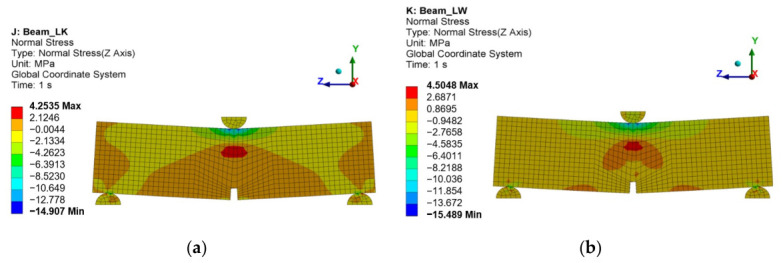
Normal stress in concrete upon destruction: (**a**) LK, (**b**) LW.

In order to determine the flexural strength of the analysed beams, force–deflection (Figure 18a) and force–notch opening (Figure 18b) under load curves were plotted. The modelling results revealed that the LW concrete beam 2 had been characterised by higher strength (17.5 kN) than the LK concrete beam 1 (17.2 kN) before material softening. The destruction of beam 1 occurred at the tensile stress of 4.25 MPa (Figure 19a). The destruction of beam 2 reinforced with steel fibres occurred at the tensile strength of 4.5 MPa (Figure 19b). It should be noted that the obtained values are greater than the limit strength of lightweight concrete (4.1 MPa) determined under uniaxial tension.

**Figure 20:**
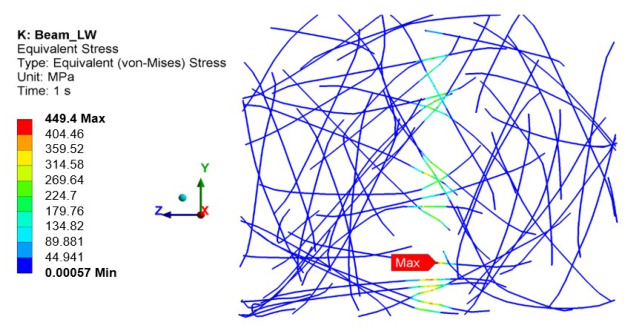
The von Mises stress in the LW beam steel fibres.

Concrete cracking exceeded the strength of the steel fibres, as shown in Figure 20.

The presented modelling approach does not reflect the actual energy dissipation mechanisms. The Menetrey–Willam MW constitutive model for concrete, described in Section 3.5.1, assumes physical nonlinearity and permanent deformation of concrete under the influence of tensile and compressive stresses. This model does not incorporate concrete damage mechanisms (notch opening). Future work plans to incorporate other constitutive models that account for concrete damage mechanisms.

The damage assessment utilised a criterion related to the properties of steel fibres. It was assumed that damage would occur when the stresses in the steel fibres across the entire cross-section of the specimen exceeded the yield strength of steel fibres. Numerical calculations were performed for deflections of 0.16 mm and 0.12 mm, where the steel stresses were observed to exceed the yield strength.

## 4. Conclusions

Lightweight concrete reinforced with steel fibres, intended for construction purposes, was designed and made as part of this study. The essential test for assessing the effectiveness of lightweight concrete reinforcement is the residual flexural tensile strength test, in which the concrete achieved high residual strength, and the fibres transferred further loads after the cement matrix cracked. The high tensile strength of lightweight fibre-reinforced concrete is caused by a uniform fibre distribution in the concrete, with no fibre clusters, as confirmed by computer tomography.

Analysing the results of strength tests, it was found that the differences between LW and LK concretes are not significant. However, taking into account the simple acceptance rule, the results of lightweight concrete tests indicate that the addition of 20 kg/m^3^ of steel fibres to lightweight concrete increases compressive strength by approximately 5%. For the flexural strength test, LW concrete achieved approximately 4% higher flexural strength than LK concrete, which opens up prospects for the use of LW concrete in structural concrete. The results of the elastic modulus and splitting tensile strength for LK and LW concretes are similar. In the study of lightweight concrete microstructure, good coverage of the porous, lightweight aggregate by the cement slurry was observed. The structure of the lightweight aggregate–cement slurry contact zone is testimony to proper mixing, initial saturation of the lightweight aggregate, and adequate care of the hardened concrete. The relatively high cement content in the concrete mix is the direct contributor to the formation of the shrinkage cracks observed in the cement slurry. Steel fibres added to the lightweight concrete were uniformly distributed and fully covered by the cement slurry, which enabled adequate assumptions for the material model. Fibre surface roughness areas were filled with cement slurry, additionally increasing the contact zone area and improving the strength characteristics. Fibre surface roughness can cause the formation of microcracks in the cement slurry, while the fibres limit the propagation of shrinkage cracks, which is a positive effect. An addition of steel fibres to lightweight concrete was observed to decrease the content of air macropores, which are typically not found in plain concrete (with natural aggregate) and require further analysis. Pore size distribution analysis has led to a conclusion that addition of fibres to lightweight concrete is beneficial because it promotes the formation of a greater number of smaller air voids, which positively affects the frost resistance of the cement matrix and the concrete. The results of experimental works and numerical simulations revealed that the flexural strength of the LW concrete containing steel fibres is ca. 5% higher than that of the LK control concrete without fibres. Based on preliminary results obtained with the numerical model of concrete reinforced with steel fibres, the authors can continue numerical work to establish the optimal distribution and content of various fibres for specific construction needs. Conducting a series of numerical simulations supported by microscopic observations across various fibre configurations will provide an in-depth understanding of the complex interactions between the fibres and the cement matrix in concrete. There are also plans to use other constitutive models in numerical simulations to evaluate the strength of LW concrete. The MicroPlane model (of numerous planes) is among those used for describing progressive cracking. In future studies, the model shall be used to optimise the material’s mechanical characteristics.

## Figures and Tables

**Table 1:** Characteristics of sintered lightweight aggregate, 1–4 mm fraction.

Characteristics	1–4 mm Fraction
Loose bulk density [kg/m^3^]	650
Chloride content [%]	≤0.05
Acid-soluble sulphates [%]	≤1.0
Total sulphur [%]	≤1.0
Radioactive radiation	I ≤ 1
Released heavy metals	Below the highest acceptable value

**Table 2:** Characteristics of sintered lightweight aggregate, 4–9 mm fraction.

Characteristics	4–9 mm Fraction
Grain density [kg/m^3^]	1300
Absorbability after 24 h [%]	20
Loose bulk density [kg/m^3^]	700
Dust content [%]	1.9
Crush resistance [MPa]	5.0

**Table 3:** Formula of lightweight concrete with steel fibres.

Ingredient	Content [kg/m^3^]
CEM I 42.5 N cement	420
Lightweight aggregate 1–4 mm	200
Lightweight aggregate 4–9 mm	610
Sand 0–2	400
Water (*)	115
Admixture-plasticiser	1% mc
Air-entraining admixture	0.25% mc
Steel fibres	20

(*) additional water for aggregate initial soaking—15% of lightweight aggregate weight (121.5 kg/m^3^).

**Table 4:** Characteristics of the LK and LW concrete mixes.

Concrete Identification	Consistency, Slump Test [mm]	Air Content [%]	Concrete Mix Density [kg/m^3^]
LK concrete (control)	70	6.5	1850
LW concrete (with fibres)	60	7.0	1875

**Table 5:** Characteristics of LK and LW fibre-reinforced concrete.

Concrete Identification	Compressive Strength After 28 Days [MPa]	Flexural Tensile Strength After 28 Days [MPa]	Splitting Tensile Strength After 28 Days [MPa]	Modulus of Elasticity in Compression After 90 Days [GPa]
LK concrete (control)	49.4 ± 2.0	7.1 ± 0.4	4.1 ± 0.2	26.8 ± 1.0
LW concrete (with fibres)	51.5 ± 2.0	7.4 ± 0.4	4.1 ± 0.2	26.2 ± 1.0

**Table 6:** Test results for residual flexural tensile strength.

Specimen Identification	Residual Flexural Tensile Strength [MPa]				
	At the Limit of Proportionality LOP f_f/ct,L_	f_R,1_	f_R,2_	f_R,3_	f_R,4_
		CMOD_1_ = 0.5 mm	CMOD_2_ = 1.5 mm	CMOD_3_ = 2.5 mm	CMOD_4_ = 3.5 mm
LW_1	4.10	2.64	3.31	3.38	3.44
LW_2	4.49	3.48	4.02	4.05	3.64
LW_3	3.89	2.92	3.39	3.39	3.19
LW_4	4.46	4.97	6.16	6.13	5.70
LW_5	4.73	5.32	6.21	6.20	5.48
LW_6	4.17	3.85	4.38	4.28	4.00
LW_7	4.42	4.41	5.03	4.58	4.21
LW_8	4.32	3.44	4.01	3.78	3.59
mean value	4.32 ± 0.34	3.88 ± 0.18	4.56 ± 0.15	4.47 ± 0.16	4.16 ± 0.17

**Table 7:** Pore size distribution in concrete.

Property	Sample	
	LK	LW
total porosity [%]	8.73	1.33
number of analysed pores	685	983
Pore Diameter [µm]		
minimum	2.5	2.5
maximum	2206	1366
average	47.6	26.5
median	9.2	9.8

**Table 8:** Material parameters for concrete with Menetrey–Willam base.

Parameter Name	Symbol (Unit)	Value
Isotropic elasticity		
Young’s Modulus	E (GPa)	26.8
Poisson’s Ratio	-	0.2
Menetrey–Willam Base		
Uniaxial compressive strength	f_c_ (MPa)	49.4
Uniaxial tensile strength	f_ct_ (MPa)	4.1
Biaxial compressive strength	f_bc_ (MPa)	50.0
Dilatancy angle	ψ (degree)	30
Plastic strain at uniaxial compressive strength	(-)	0.001
Relative stress at the beginning of non-linear hardening	(-)	0.4
Residual compressive relative stress	(-)	0.2
Plastic strain limit tension	(-)	0.01
Residual tensile relative stress	(-)	0.2

**Table 9:** Material parameters for steel fibres.

Parameter	Symbol (Unit)	Value
Isotropic elasticity		
Young’s modulus	E (GPa)	210.0
Poisson’s ratio	-	0.3
Bilinear isotropic hardening		
Yield strength	E (MPa)	250
Tangent modulus	E (MPa)	6894.8

## Data Availability

The original contributions presented in this study are included in the article. Further inquiries can be directed to the corresponding author.

## Abbreviations

The following abbreviations are used in this manuscript:

LWAC	Lightweight aggregate concrete
LK	Lightweight control concrete with no fibres
LW	Lightweight concrete with fibres
